# Hybrid Organic–Si C-MOSFET Image Sensor Designed with Blue-, Green-, and Red-Sensitive Organic Photodiodes on Si C-MOSFET-Based Photo Signal Sensor Circuit

**DOI:** 10.3390/nano14131066

**Published:** 2024-06-21

**Authors:** Ui-Hyun Jeong, Joo-Hyeong Park, Ji-Ho Choi, Woo-Guk Lee, Jea-Gun Park

**Affiliations:** 1 Department of Electronic Engineering, Hanyang University, Seoul 04763, Republic of Korea; 2 Samsung Advanced Institute of Technology, 129, Samsung-ro, Yeongtong-gu, Suwon-si 16677, Republic of Korea; 3 Department of Nanoscale Semiconductor Engineering, Hanyang University, Seoul 04763, Republic of Korea; 4 Advanced Semiconductor Materials & Devices Development Center, Hanyang University, Seoul 04763, Republic of Korea

**Keywords:** CMOS image sensor, organic photodiode, image sensor pixel

## Abstract

The resolution of Si complementary metal–oxide–semiconductor field-effect transistor (C-MOSFET) image sensors (CISs) has been intensively enhanced to follow the technological revolution of smartphones, AI devices, autonomous cars, robots, and drones, approaching the physical and material limits of a resolution increase in conventional Si CISs because of the low quantum efficiency (i.e., ~40%) and aperture ratio (i.e., ~60%). As a novel solution, a hybrid organic–Si image sensor was developed by implementing B, G, and R organic photodiodes on four n-MOSFETs for photocurrent sensing. Photosensitive organic donor and acceptor materials were designed with cost-effective small molecules, i.e., the B, G, and R donor and acceptor small molecules were Coumarin6 and C_60, DMQA and MePTC, and ZnPc and TiOPc, respectively. The output voltage sensing margins (i.e., photocurrent signal difference) of the hybrid organic–Si B, G, and R image sensor pixels presented results 17, 11, and 37% higher than those of conventional Si CISs. In addition, the hybrid organic–Si B, G, and R image sensor pixels could achieve an ideal aperture ratio (i.e., ~100%) compared with a Si CIS pixel using the backside illumination process (i.e., ~60%). Moreover, they may display a lower fabrication cost than image sensors because of the simple image sensor structure (i.e., hybrid organic–Si photodiode with four n-MOSFETs).

## 1. Introduction

**Figure 1:**
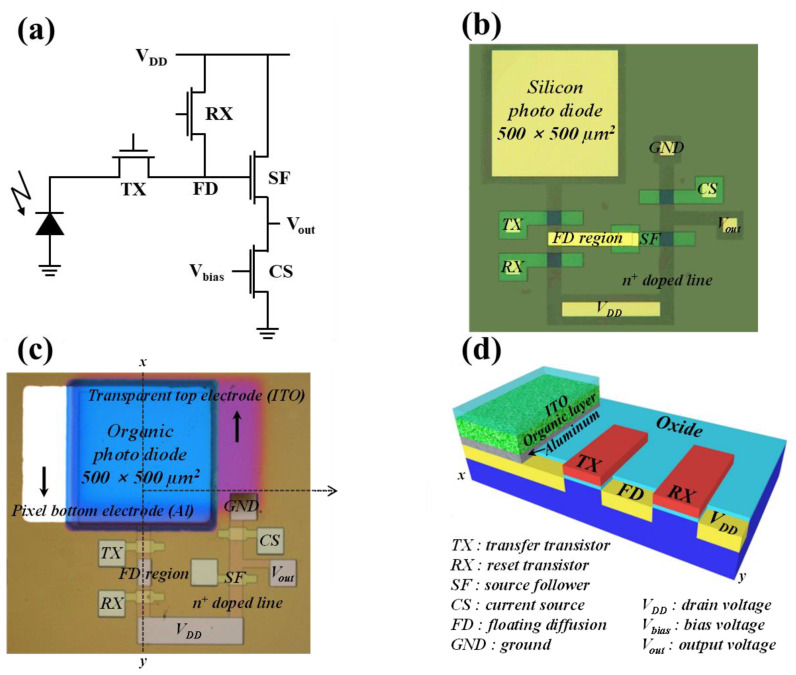
Design of hybrid organic Si image sensor pixels. (**a**) Pixel design of an organic or Si photo diode and a sensing circuit using 4 n-MOSFETs CIS, (**b**) optical microscopic top-view image of a Si CIS pixel, (**c**) optical microscopic top-view image of hybrid organic Si R-image sensor pixel, and (**d**) cross sectional view of hybrid organic Si R-image sensor.

Recently, the pixel resolution of complementary metal–oxide–semiconductor field-effect transistor (MOSFET) image sensors (CISs) has been continuously increased for cellular phones and electric automotive vehicles up to 200 million pixels [1,2,3,4,5,6], decreasing the size of a single pixel on the image sensor to 0.56 μm. In general, the pixel size of Si CISs has been scaled down to less than a micron, which results in significant cross-talk among the blue (B), green (G), and red (R) color signals and significantly decreases the signal-to-noise ratio (SNR) due to the limited number of absorbed photons through B, G, and R pixels, as shown in Figure 1a. Consequently, this decreases the SNR. In addition, the scaling down of the pixel size has been limited by the diffraction limit of the optical system. Thus, research on enhancing the efficiency of absorbing photo light with the Si photodiodes of conventional CISs has been intensively conducted to achieve extremely high pixel resolution; this includes structural changes in the CIS from frontside illumination (FSI) to backside illumination (BSI) [7,8,9,10,11] and research on the structure and process of doped silicon photodiodes [12,13]. These studies showcase the ongoing efforts to improve the diffraction limit and SNR by increasing the size of the photodiode and the number of electrons that can be generated by light for the same pixel size. Note that FSI has a photo signal sensor circuit on the Si photodiodes of B, G, and R pixels, while BSI implements a photo signal sensor circuit on the Si photodiodes of B, G, and R pixels, as shown in Figure S2. It has been reported that CISs using a BSI structure achieve ~40% higher sensitivity than CISs using FSI [14,15,16,17]. Although the resolution of pixels for CISs using BSI has been scaled down steadily, the SNR improvement based on BSI has been decreased due to the cross-talk enhancement among photo B, G, and R color signals, which was resolved by introducing Deep-Trench Isolation (DTI) among B, G, and R pixels [18]. However, to achieve a further high resolution of >200 million pixels, the aperture ratio in current CISs using BSI has a limit of ~60% since the Si photodiode and sensor circuit are located in parallel. As an alternative solution to overcoming the limits on the aperture ratio, an organic image sensor fabricated on glass has been proposed, since B-, G-, and R-sensitive organic layers could cover all areas of the B, G, and R pixels, demonstrating an ideal ~100% aperture ratio [19,20,21,22,23,24,25,26,27]. However, it is difficult to apply this organic image sensor with a higher resolution of 200 million, since the scaling down of the organic transistors for the sensor circuit cannot approach dimensions smaller than a sub-micrometer. Thus, in our study, a novel hybrid organic–Si B, G, and R image sensor was designed to overcome the aperture ratio limits in current CISs using BSI (i.e., ~60%) and was able to achieve an ideal ~100% aperture ratio, as shown in Figure 1b. Hybrid organic–Si B, G, and R image sensor pixels were fabricated with a vertical structure of B, G, and R color filters; B, G, and R organic photodiodes; and a Si sensor circuit using four n-MOSFETs. In addition, the B, G, and R organic photodiodes were designed by using cost-effective small-molecule donor and acceptor layers, i.e., Coumarin6 and C_60, DMQA and MePTC, and ZnPc and TiOPc for the B, G, and R organic donor and acceptor layers. Note that the hybrid organic–Si B, G, and R image sensor pixels could achieve an aperture ratio of ~100%, since the B-, G-, and R–sensitive organic layers can cover all areas of B, G, and R pixels, as shown in Figure S1b. Moreover, the quantum efficiency of Si photodiodes for CISs using BSI is limited to rates of up to ~40% [28], and the quantum efficiency of the B, G, and R organic photodiodes in the proposed hybrid organic–Si B, G, and R image sensor pixels should be higher than ~40%, which will be proved later. Furthermore, the proposed hybrid organic–Si B, G, and R image sensor pixels have a low CIS fabrication cost, since B, G, and R organic photodiodes can be implemented on a conventional photo signal sensor circuit using four n-MOSFETs, as shown in Figure 1b. As a reminder, the CIS using BSI was fabricated via the formation of logic circuits and Si photodiodes connected with four n-MOSFETs, which were assembled like traditional image sensors and color filters through various processes, as shown in Figure S2. In our study, the dependencies of the material properties (i.e., absorption and energy band gap) on the B, G, and R organic donor and acceptor layers were investigated to understand how the organic donor layer generates excitons and electrons. In addition, a Si CIS pixel and hybrid organic–Si B, G, and R image sensor pixels were fabricated to compare the voltage sensing margin differences (i.e., photocurrent signal difference) in the image sensor, and the voltage sensing margin differences were examined as functions of the Si CIS pixel and hybrid organic–Si B, G, and R image sensor pixels. Finally, the B–G and G–R light cross-talk performance was estimated between the Si CIS and the hybrid organic–Si B, G, and R image sensor.

## 2. Results

### 2.1. Design of Hybrid Organic–Si B, G, and R Image Sensor Pixels

Hybrid organic–Si B, G, and R image sensor pixels were designed with B, G, and R organic–Si photodiodes and four n-MOSFETs (TX: transfer transistor; RX: reset transistor; SF: source follower transistor; and CS: current source transistor), where the circuit of a hybrid B, G, and R pixel is presented like a conventional circuit of a Si photodiode, as shown in Figure 1a. As a reference, a Si pixel was also produced with four n-MOSFETs, as shown in Figure 1b. Particularly, an R hybrid organic–Si photodiode was fabricated with the vertical structure of a top ITO electrode, an R organic donor layer, an R organic acceptor layer, a bottom Al electrode, and an n+ Si substrate, which were connected with four n-MOSFETs (i.e., TX, RX, SF, and CS); the area of the R hybrid organic–Si photodiode was 500 × 500 um^2^, as shown in Figure 1c,d. Remember that the ITO electrode is a transparent material, and the Al electrode has good ohmic properties against the n+ Si substrate.

### 2.2. Optical Properties and Energy Band Diagrams of B, G, and R Organic–Si Photodiodes

**Figure 2:**
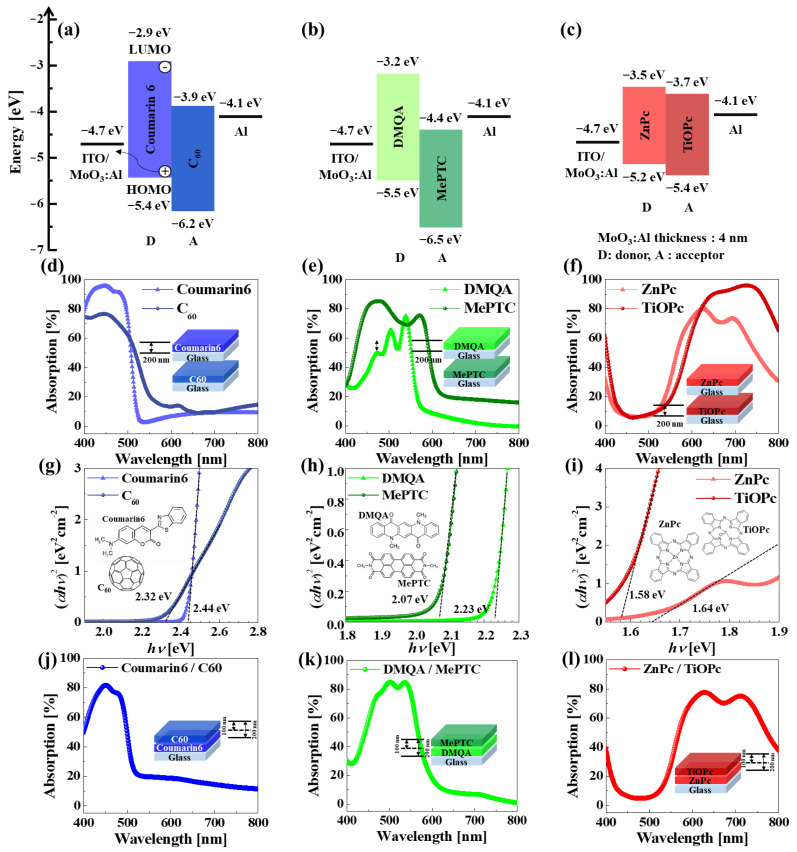
Optoelectronic properties of B-, G-, and R-sensitive organic donor and acceptor small molecules. Schematic energy band diagram of organic photodiodes for (**a**) blue, (**b**) green, and (**c**) red. Absorption of small-molecule donor and acceptor layers for (**d**) blue (i.e., Coumarin6 and C_60), (**e**) green (i.e., DMQA and MePTC), and (**f**) red (i.e., ZnPc and TiOPc). Energy band gap of small-molecule donor and acceptor layers for (**g**) blue (i.e., Coumarin6 and C_60), (**h**) green (i.e., DMQA and MePTC), and (**i**) red (i.e., ZnPc and TiOPc). Absorption of mixed (bi-layer) small-molecule donor and acceptor layers for (**j**) blue (i.e., Coumarin6/C_60), (**k**) green (i.e., DMQA/ MePTC), and (**l**) red (i.e., ZnPc/TiOPc).

To estimate the photocurrent of the B, G, and R organic–Si photodiodes, the optical properties, such as the absorption and energy band gap of the B, G, and R organic bi-layers (i.e., donor and acceptor: D/A), were investigated, where the bi-layer structure and the thickness of the bi-layers were optimized to achieve the maximum optical properties. In general, Coumarin6, a B-sensitive organic material, is known as a donor material in small-molecule solar cells, emitters of organic light-emitting diodes [29,30], laser dyes, and sensitizers of dye-sensitized solar cells because of its high stability [31,32], as well as lower production cost, as shown in Figure 2a. Moreover, C_60 has been reported as a good electron acceptor [33]. In addition, DMQA, a G-sensitive organic donor material, is well known as a fluorescent dye [34,35,36], and MePTC has been reported as an n-type acceptor organic material, showing high thermal stability [37], as shown in Figure 2b. ZnPc is known as a red donor material [38], presenting good transport properties and high absorption, and TiOPc has been reported as a red acceptor material [39] with high hole mobility and optical band gap, as shown in Figure 2c. Moreover, the B, G, and R organic–Si photodiodes using the presented organic materials have a relatively simple fabrication process for organic CISs compared with the conventional fabrication process of BSI [40]. To estimate the optical properties (i.e., absorptions and energy band gap) of 200 nm thick B-, G-, and R-sensitive organic donor and acceptor layers, B, G, and R donor and acceptor layers were evaporated on glass. As shown in Figure 2a–c, the absorption wavelength and the maximum absorption of the B donor layer (i.e., coumarin6) were 400~530 nm and 95.6%, respectively, as shown in Figure 2d. The absorption wavelength and maximum absorption of the B acceptor layer (i.e., C_60) were 400~545 nm and 76.6%, respectively, as shown in Figure 2d. These absorption results indicate that the absorption of the B donor layer stacked on the acceptor layer (i.e., B-sensitive organic bi-layer) is determined by the B acceptor layer. In addition, the absorption wavelength and maximum absorption of the G donor layer (i.e., DMQA) were 425~575 nm and 75%, respectively, as shown in Figure 2e. The absorption wavelength and maximum absorption of the G acceptor layer (i.e., MePTC) were 400~620 nm and 85.25%, respectively, as shown in Figure 2e. These absorption results imply that the absorption of the G donor layer stacked on the acceptor layer (i.e., G-sensitive organic bi-layer) is determined by the G donor layer. Moreover, the absorption wavelength and maximum absorption of the R donor layer (i.e., ZnPC) were 530~800 nm and 79.9%, respectively, as shown in Figure 2f. The absorption wavelength and maximum absorption of the R acceptor layer (i.e., TiOPc) were 540~800 nm and 95.9%, respectively, as shown in Figure 2f. These absorption results mean that the absorption of the R donor layer stacked on the acceptor layer (i.e., R-sensitive organic bi-layer) is determined by the R donor layer. The energy band gaps of the B-sensitive organic donor (i.e., coumarin6) and acceptor layers (i.e., C_60) were 2.44 and 2.32 eV, respectively, as shown in Figure 2g. In addition, the energy band gaps of the G-sensitive organic donor (i.e., DMQA) and acceptor layers (i.e., MePTc) were 2.23 and 2.07 eV, respectively, as shown in Figure 2h. Moreover, the energy band gaps of the R-sensitive organic donor (i.e., ZnPc) and acceptor layers (i.e., TiOPc) were 1.64 and 1.58 eV, respectively, as shown in Figure 2i [41,42,43]. It was reported that the work functions of ITO and Al were −4.7 and −4.1 eV; the lowest unoccupied molecular orbital (LUMO) levels of B (i.e., Coumarin6), G (i.e., DMQA), and R (i.e., ZnPc) donor layers were −2.9, −3.2, and −3.5 eV; and the highest unoccupied molecular orbital (HUMO) levels of B (i.e., C_60), G (i.e., MePTC), and R (i.e., TiOPc) acceptor layers were −6.2, −6.5, and −5.4 eV, respectively [44,45,46,47,48,49,50]. Based on the energy band gaps of B-, G-, and R-sensitive organic donor and acceptor layers, energy band diagrams of the B, G, and R organic photodiodes were drawn, respectively, as shown in Figure 2a–c. As is well known, B-, G-, and R-sensitive organic donors can absorb B, G, and R light, respectively, generating excitons that are transferred toward the interface between organic donors and acceptors and separated into electrons and holes at the interface. Thus, the generated holes drift toward the ITO top electrode, while the generated electrons drift toward the bottom Al electrode, producing photocurrent. Finally, the absorption of the B, G, and R donor and acceptor bi-layers was evaluated by evaporating 100 nm thick B, G, and R donor and acceptor bi-layers on glass. The absorption wavelength and maximum absorption of the B donor and acceptor bi-layers (i.e., Coumarin6 and C_60) were 400~520 nm and 81.7%, respectively, as shown in Figure 2j. In addition, the absorption wavelength and maximum absorption of the G donor and acceptor bi-layers (i.e., DMQA and MePTc) were 415~615 nm and 84.8%, respectively, as shown in Figure 2k. Moreover, the absorption wavelength and maximum absorption of the R donor and acceptor bi-layers (i.e., ZnPC and TiOPc) were 525~800 nm and 77.6%, respectively, as shown in Figure 2l. According to the comparison in Figure 2j–l, the absorption of the photosensitive organic donor and acceptor bi-layers was ordered from high to low values as G (i.e., 84.8%), B (i.e., 81.7%), and R (i.e., 77.6%).

### 2.3. Dependency of Voltage Sensing Margin on Light Illumination Intensity for Hybrid Organic–Si G Image Sensor Pixel

**Figure 3:**
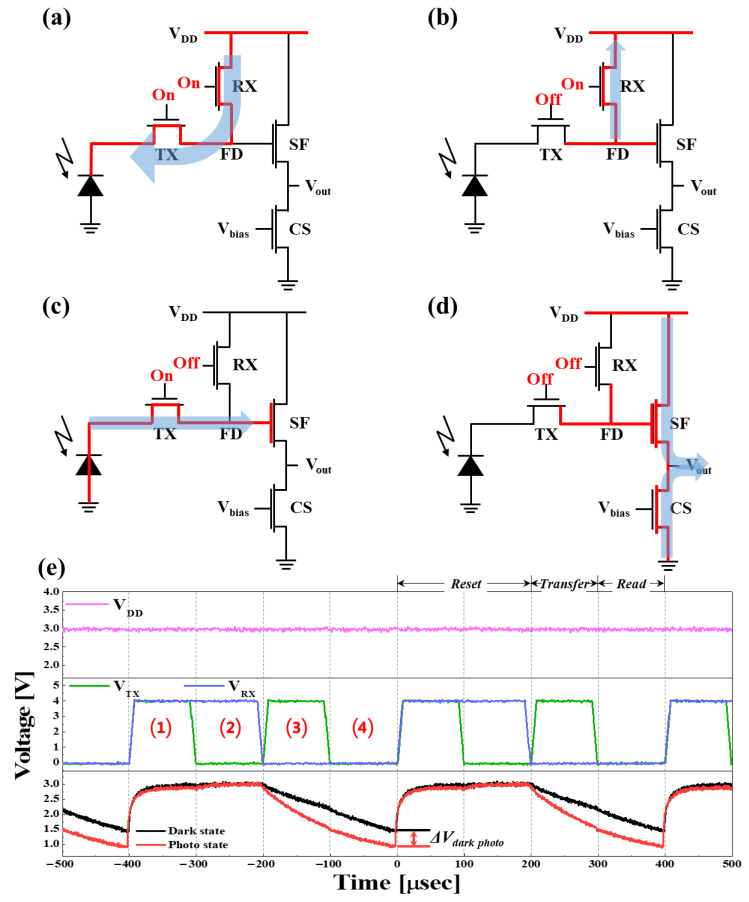
Operation mechanism of the CIS pixel fabricated with a Si photodiode and four n-MOSFETs. (**a**) Voltage transfer from *V_DD_* in 0–100 μsec to bottom electrode. (**b**) Floating diffusion region reset in 100–200 μsec, (**c**) electron transport to floating diffusion region from B, G, and R hybrid organic–Si photodiodes in 200–300 μsec, (**d**) floating diffusion region floating and sensing the output voltage in 300–400 μsec, and (**e**) input voltage pulse of *V_DD_*, pulses of *TX* and *RX* of CIS cells, and output voltage sensing margin (Δ*V_out_*). (1)~(4) present the input pulses of *TX* and *RX* in (**a**–**d**) respectively.

To confirm the feasibility of a Si pixel being operated by an *n*+*p* Si photodiode and four n-MOSFETs (i.e., TX, RX, SF, and CS), the voltage sensing margin of the *n*+*p* Si photodiode of a Si pixel was estimated. Initially, pulse voltages of 4 V with a pulse width of 100 μs were applied to the gates of both TX and RX to turn on both RX and TX, while a constant voltage of 3 V was applied to *V_DD_*, which was connected with the draining of RX. When both TX and RX were turned on, the *V_DD_* of 3 V was transferred to the bottom electrode of the photodiode, as shown in Figure 3a and represented by (1) in Figure 3e. Secondly, pulsed voltages of 0 and 4 V were applied to the gates of TX and RX, respectively. TX was turned off and RX turned on, resetting the floating diffusion (FD) region of 3 V, as shown in Figure 3b and represented by (2) in Figure 3e. Thirdly, pulsed voltages of 4 and 0 V were applied to the gates of TX and RX, respectively. TX was turned on and RX turned off, with the transport being generated by the *n*+*p* Si photodiode toward the FD region, as shown in Figure 3c and represented by (3) in Figure 3e. Finally, TX and RX pulse voltages of 0 V were applied to the gates of both TX and RX. Both TX and RX were turned off, thereby sensing the output voltage (*V_out_*) since the voltage of the FD region corresponding to the generated electron amount was applied to the SF gate, where CS was then turned on. To confirm the operation of the Si CMOS image sensor pixel, the voltage change in the FD region was measured by suppling the pulse voltage of TX and RX, according to the operational logic of a Si pixel, as described in Figure 3a–d, the top picture of *V_DD_* in Figure 3e, and the middle picture in Figure 3e. Without exposing the *n*+*p* Si photodiode to light, with TX and RX turned on, the voltage of the FD region was 3 V, as represented by (1) in Figure 3e and by the dark line of the bottom picture in Figure 3e. Afterwards, TX was turned off, while RX was turned on, and the voltage of the FD region was also 3 V, as represented by (2) in Figure 3e and by the dark line in the bottom picture of Figure 3e. Then, TX was turned on and RX turned off, and the voltage of the FD region was reduced from 3 to 2 V in 100 μs, as represented by (3) in Figure 3e and by the dark line in the bottom picture of Figure 3e. Sequentially, when both TX and RX were turned off, the voltage of the FD region decreased from 2 to 1.5 V, as represented by (4) in Figure 3e and by the dark line in the bottom picture of Figure 3e. Thus, the voltage difference in the FD region, called the voltage sensing margin (*V_out_*) of a Si CMOS image sensor pixel, was 1.5 V, corresponding to how many electrons were produced at the *n*+*p* Si photodiode by absorbing photo light. However, when using a white light intensity of 20,000 lux and suppling sequential pulses of TX and RX as described in the middle picture in Figure 3e, the voltage of the FD region was 2.0 V, meaning that the voltage sensing margin of the pixel was 2.0 V, as represented by the red bottom line in Figure 3e. The difference in the voltage sensing margin (*V_out_*) was defined as the voltage sensing margin without and with white-light illumination. As a result, ∆*V_out_* was 0.5 V, meaning that the voltage of the FD region increased by 0.5 V due to the electrons generated by exposing the *n*+*p* Si photodiode to white light. Thus, our designed Si CMOS image sensor pixel in Figure 1b could demonstrate evident photosensitivity (i.e., voltage sensing margin difference: ∆*V_out_*) and be used for estimating a photosensitivity difference between the Si CMOS image sensor pixel in Figure 1b and the hybrid organic–Si B, G, and R image sensor pixel in Figure 1c.

**Figure 4:**
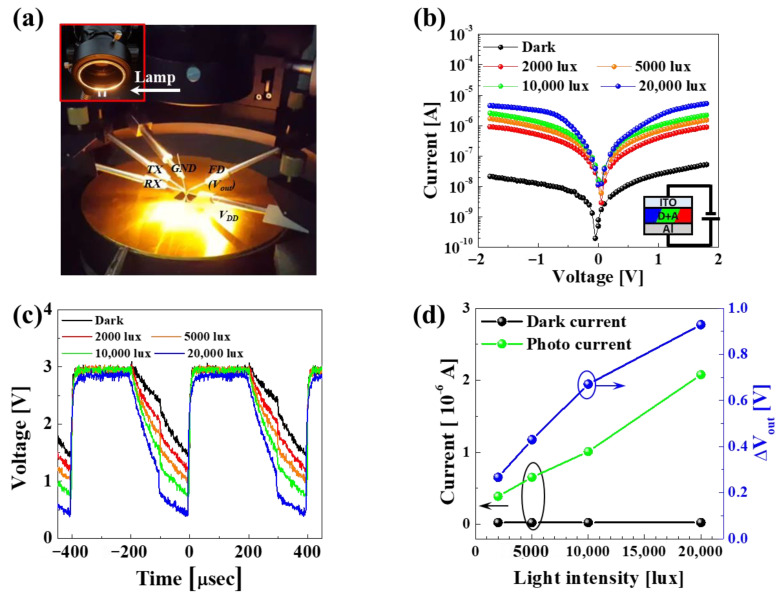
Dependency of photo signal sensitivity on hybrid organic–Si G image sensor pixel. (**a**) Probe measurement system under light illumination from white lamp, (**b**) dark current and photocurrents vs. applied voltage for G hybrid organic–Si photodiode, (**c**) dependency of voltage sensing margin on light illumination intensity for hybrid organic–Si G image sensor pixel, and (**d**) correlation between photocurrent of G hybrid organic–Si photodiode and voltage sensing margin of hybrid organic–Si G image sensor pixel. The graph values inside each circle correspond to the axis indicated by the arrow.

To confirm the proper operation of the proposed G hybrid organic–Si photodiode, the influence of the light illumination intensity on current vs. voltage (I–V) was investigated, where variable voltages (i.e., −2~+2 V) were applied between the top ITO and bottom Al electrodes under variable light illumination intensity (i.e., 0~20,000 lux), as shown in Figure 4a and Video S1. In the dark state (i.e., without light illumination), the dark current linearly increased from 4.97 × 10^−10^ to 5.27 × 10^−8^ A when the applied bias increased from 0 to 1.8 V, since the organic donor and acceptor behave as an organic resistance layer. Thus, we obtained a symmetrical I–V curve when the applied voltage spanned from 0 to 1.8 V and from 0 to –1.8 V. On the other hand, in the photo state (i.e., with light illumination), the photocurrent was significantly enhanced when light with an intensity of 2000 lux was directed at the G hybrid organic–Si photodiode. This resulted in a symmetrical I–V curve to scanning positive and negative applied voltage, since a mixed layer of the G organic donor (i.e., DMQA) and acceptor (i.e., MePTC) was produced upon evaporating the G organic donor on the G organic acceptor. The absorbed photons in the organic donor and acceptor mixture layer produced excitons. Then, the excitons separated into electrons and holes, making the electrons drift toward the bottom Al electrode and the holes toward the top ITO electrode under scanning positive applied voltage or vice versa under scanning negative applied voltage. In addition, the photocurrent of the G hybrid organic–Si photodiode at an applied voltage of 1 V increased linearly from 3.89 × 10^−7^ to 2.08 × 10^−6^ when the light illumination intensity increased from 2000 to 20,000 lux, as shown in Figure 4d. Moreover, the voltage sensing margin of the hybrid organic–Si G image sensor pixel was estimated as a function of the light illumination intensity, as shown in Figure 4c. In the dark state (i.e., without light illumination), the voltage sensing margin was 1.40 V. The operations of applying voltage pulse sequences of *V_DD_*, *V_TX_*, and *V_RX_* are shown in Figure 3e. Otherwise, the voltage sensing margin increased from 1.18 to 0.45 V, and the light illumination intensity was increased from 2000 to 20,000, as shown in Figure 4c. The voltage sensing margin of a hybrid organic–Si G image sensor pixel was relatively well correlated with the photocurrent of the G hybrid organic–Si photodiode, demonstrating that the mixed organic donor (i.e., DMQA) and acceptor (i.e., MePTC) could produce excitons readily via absorbing the white light generated by a halogen lamp; the generated excitons were separated into electron and hole pairs at the interface between donor and acceptor molecules, and the separated electrons and holes drifted toward the electrodes.

### 2.4. Difference in Voltage Sensing Margins with and without White-Light Illumination for Hybrid Organic–Si B, G, and R Image Sensor Pixel 

**Figure 5:**
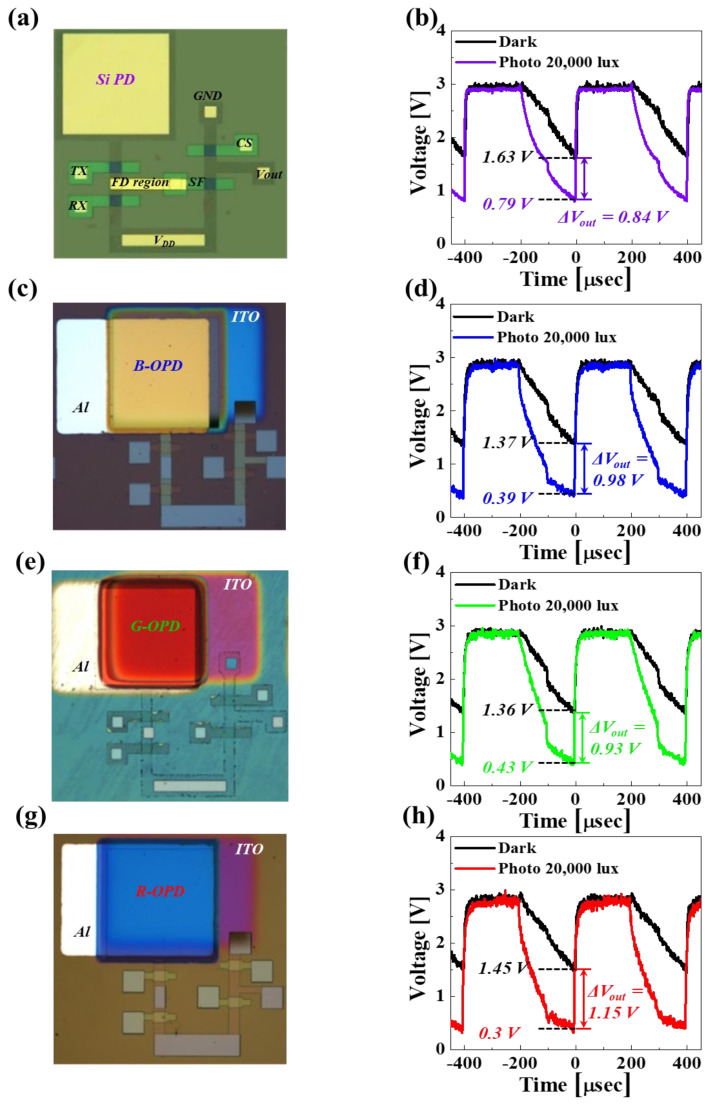
Voltage sensing margin difference (i.e., photo signal sensing difference) for hybrid organic–Si B, G, and R image sensor pixels. (**a**) Optical microscope image of Si photodiode, (**b**) Δ*V_out_* of Si CIS cell, (**c**) optical microscope image of blue-sensitive organic–Si photodiode, (**d**) Δ*V_out_* of blue-sensitive organic CIS cell, (**e**) optical microscope image of G hybrid organic–Si photodiode, (**f**) Δ*V_out_* of green-sensitive organic CIS cell, and (**g**) optical microscope image of red-sensitive organic–Si photodiode, and (**h**) Δ*V_out_* of red-sensitive organic CIS cell.

To estimate the merit of hybrid organic–Si B, G, and R image sensor pixels compared with the Si image sensor pixel, the voltage sensing margin differences in the image sensor pixels were measured with and without white-light illumination, with a light intensity of 20,000 lux. For the Si image sensor pixel, fabricated with a Si photodiode and four n-MOSFETs (i.e., TX, RX, SF, and CS), as shown in Figure 5a, the difference in the voltage sensing margin with (0.79 V) and without (1.63 V) white-light illumination was 0.84 V, where the VDD, TX, and RX applied voltages were 3, 4, and 4 V, as shown in Figure 5b. Further, for the hybrid organic–Si B image sensor pixel, fabricated with a B organic–Si photodiode (i.e., transparent, 50 nm thick top ITO electrode/mixed B organic layer of 150 nm thick donor and 100 nm thick acceptor/bottom ohmic Al electrode) and four n-MOSFETs, as shown in Figure 5c, the difference in the voltage sensing margin with (0.39 V) and without (1.37 V) white-light illumination was 0.98 V, as shown in Figure 5d. Note that the color of the B hybrid organic–Si photodiode was yellow since B light was absorbed, while G and R light was reflected from the bottom Al electrode. In addition, for the hybrid organic–Si G image sensor pixel, fabricated with a G organic–Si photodiode (i.e., top ITO electrode/mixed G organic layer of 175 nm thick donor and 100 nm thick acceptor/bottom Al electrode) and four n-MOSFETs, as shown in Figure 5e, the difference in the voltage sensing margin with (0.43 V) and without (1.36 V) white-light illumination was 0.93 V, as shown in Figure 5f. Note that the color of the G hybrid organic–Si photodiode was red since G light was absorbed, while B and R light was reflected from the bottom Al electrode. Moreover, for the hybrid organic–Si R image sensor pixel, fabricated with an R organic–Si photodiode (i.e., top ITO electrode/mixed R organic layer of 175 nm thick donor and 100 nm thick acceptor/bottom Al electrode) and four n-MOSFETs, as shown in Figure 5g, the difference in the voltage sensing margin with (0.3 V) and without (1.45 V) white-light illumination was 1.15 V, as shown in Figure 5h. Furthermore, the color of the R hybrid organic–Si photodiode was blue since R light was absorbed, while B and G light was reflected from the bottom Al electrode. By comparing Figure 5b,d,f,g, we can see that the differences in the voltage sensing margin of B (0.98 V), G (0.93 V), and R (1.15 V) hybrid organic–Si image sensor pixels were 17, 11, and 37% higher than that of the Si image sensor pixel (0.84 V), implying that the quantum efficiency of the B, G, and R hybrid organic–Si photodiodes was higher than that of the Si photodiode. Note that the voltage sensing margin of the image sensor pixels decreases as the number of electrons generated in the photodiode increases, which increases the difference in the voltage sensing margin under the same light intensity. Therefore, as shown in Figure 5 and Figures S5–S7, the B, G, and R hybrid organic–Si image sensor pixels showed higher differences in voltage sensing margins and photocurrent than the Si image sensor pixel, showing that pixel sensitivity was improved. In addition, our designed hybrid organic–Si B, G, and R image sensors provide an aperture ratio of almost 100% and utilize a simple photodiode structure with frontside illumination, significantly enhancing photosensitivity and remarkably reducing the production costs of the image sensor. Moreover, compared with a previous research study titled “Organic-on-silicon complementary metal–oxide–semiconductor color image sensors”, which applied pure organic photodiodes to CMOS image sensors for specific colors, we achieved higher absorption and a comparable level of high photocurrent across all B, G, and R colors [51]. Remember that the Si image sensor fabricated with backside illumination presented an aperture ratio of ~60% and is based on an expensive bonding process between the wafer fabricated with a Si photodiode and related circuit and the wafer fabricated with the B, G, and R color filters and related circuit.

**Figure 6:**
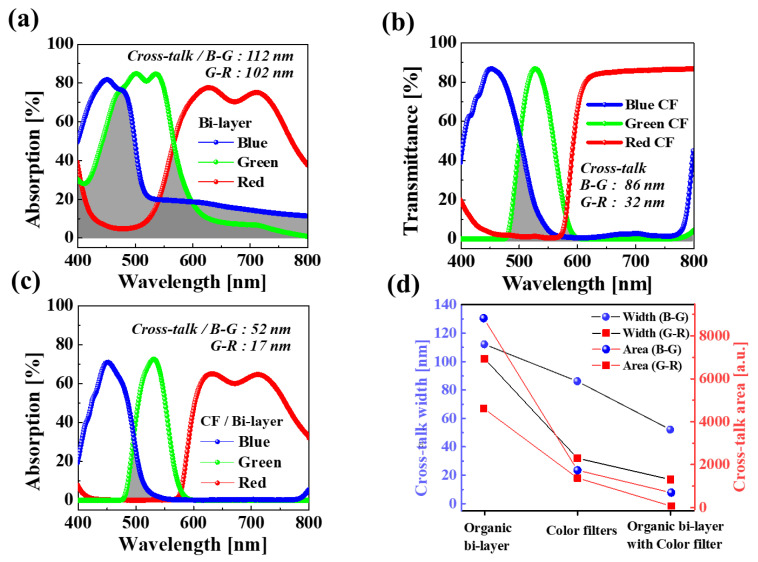
Cross-talk of B–G and G–R colors for hybrid organic–Si image sensor. (**a**) Absorbance of B, G, and R hybrid organic–Si photodiodes. (**b**) Transmittance of B, G, and R color filters used in conventional Si image sensor pixels. (**c**) Absorbance of B, G, and R hybrid organic–Si photodiodes based on B, G, and R color filters in (**b**). (**d**) Cross-talk width and area values of B–G and G–R colors for hybrid organic–Si image sensor; color filters; and hybrid organic–Si image sensor with B, G, and R color filters.

Furthermore, the hybrid organic–Si B, G, and R image sensor pixels demonstrated excellent absorbed B, G, and R color gamut due to the adoption of B, G, and R color filters, allowing for absorbed B, G, and R light cross-talk. The B–G and G–R cross-talk widths for our designed hybrid organic–Si B, G, and R image sensor pixels were 112 and 102 nm, as shown in Figure 6a. In addition, the B–G, and G–R cross-talk widths with commercial B, G, and R color filters were 86 and 32 nm in, as shown in Figure 6b. Moreover, the B–G (i.e., ~52 in width) and G–R (i.e., ~17 in width) cross-talk values for our designed hybrid organic–Si B, G, and R image sensor with vertically stacked B, G, and R color filters were considerably reduced, as shown in Figure 6c, indicating that the hybrid organic–Si B, G, and R image sensor pixels could clearly improve the color gamut of the absorbed B, G, and R light, as shown in Figure 6d.

## 3. Materials and Methods

Instead of using R, G, and B color filters in current Si CISs, three individual B, G, and R hybrid organic–Si photodiodes were designed by using photosensitive organic materials: 3–(2–Benzothiazolyl)–7–(diethylamino)coumarin (Coumarin6) and Fullerene (C_60) as B materials; 5,12–Dihydro–5,12–dimethylquino [2,3–b]acridine–7,14–dione (DMQA) and N, N′–Dimethyl–3,4,9,10–perylenedicarboximide (MePTC) as G materials; and Zinc phthalocyanine (ZnPc) and titanium oxide phthalocyanine (TiOPc) as R materials, as shown in Figure S1. In addition, for a unit cell of a CIS cell, four n-type MOSFETs, consisting of a transfer transistor (TX), a reset transistor (RX), a current source transistor (CS), and a source follower transistor (SF), were designed, as shown in Figure 1a. In particular, four types of photodiodes for CIS cells were individually fabricated on p-type Si wafers, i.e., B, G, and R hybrid organic–Si photodiodes and a conventional Si photodiode, as shown in Figure 1b,c. First, to adjust the threshold voltage (Vth) of the four transistors to 0.5 V, blanket implantation with a boron ion dose of 2 × 10^12^ atoms/cm^2^ and an acceleration voltage of 6 keV was performed. It was confirmed that the Vth of those transistors was adjusted to about 0.5 V, as shown in Figure S3. In addition, a 10 nm thick gate oxide was grown on a p-type doped Si wafer, with a concentration of 2 × 10^15^ boron atoms/cm^3^ via dry oxidation, followed by a 200 nm thick n+ doped poly-Si gate being deposited. After the gate with an area of 50 × 50 μm^2^ was patterned through photolithography and reactive ion etching (RIE), a 200 nm thick Si oxide (SiO_x_) was deposited via plasma-enhanced chemical vapor deposition (PECVD) as a mask for implanting into the source/drain and photodiode. After patterning the source and drain regions of the four transistors and the photodiode, ion implantation with an arsenic ion dose of 1 × 10^15^ atoms/cm^2^ and an acceleration voltage of 60 keV was performed. Then, the devices were subjected to rapid thermal annealing (RTA) at 1000 °C for 3 s to activate dopants, with gas annealing (FGA) at 450 °C in 2% H_2_ and 98% N_2_ ambient conditions. To define a photodiode region in a CIS cell, a 500 nm thick SiO_x_ layer was deposited by PECVD, and a photodiode region of 500 × 500 μm^2^ was patterned by using photolithography and RIE. In addition, as a bottom electrode material, aluminum (Al) was selected among four metals: aluminum (Al), gold (Au), platinum (Pt), and tungsten (W). This was because Al exhibited the lowest dark current in the reverse bias condition, resulting from the experiment on the dependency of dark currents of photodiodes on those electrode materials, as shown in Figure S4. Therefore, a 150 nm thick Al bottom electrode was deposited on the photodiode region in the CIS cell. By investigating the effect of the thickness of the donor materials on the output voltage sensing margin of CISs, the thickness of the three organic donor materials was optimized. The optimal thickness of Coumarin6 for blue was 150 nm because it showed a higher output voltage sensing margin (0.98 V) than the thickness conditions of 100 and 125 nm, showing an output voltage sensing margin of 0.19 and 0.95 in, respectively, as shown in Figure S5. In addition, for the case of DMQA for green, the 175 nm thick film showed a superior output voltage sensing margin (0.93 V) to the thickness condition of 150 nm (0.66 V), as shown in Figure S6. Moreover, for the case of ZnPc for red, the 175 nm thick film exhibited a higher output voltage sensing margin (1.15 V) than the thickness conditions of 125 (0.94 V) and 150 nm (0.97 V), respectively, as shown in Figure S7.

Three groups of donor and acceptor materials were individually deposited on the bottom electrode of the photodiode by using a thermal evaporator. Following this, as a buffer layer between the organic material and indium tin oxide (ITO), a MoO_3_:Al layer of 4 nm was co-deposited, followed by an ITO of 50 nm for the top electrode through a sputtering process under conditions of a 50 W radio frequency (RF) [52]. In addition, the optical properties of those materials in the film were investigated by using 100 nm thick single layers and 200 nm thick bi-layers of donor and acceptor materials for the blue, green, and red colors. The optical properties of those organic materials were analyzed by ultraviolet–visible (UV–Vis) spectroscopy. Moreover, the electrical characteristics of the photodiodes were determined at room temperature by using a 4155C semiconductor parameter analyzer. In particular, the output voltages of four CIS cells were analyzed by applying a direct-current (DC) bias of 3 V, as an input pulse to the gates of TX and RX. A schematic cross-sectional view of the fabricated CIS cell along with the x–y direction is shown in Figure 1d. Further, the fabricated film structures of the B, G, and R hybrid organic–Si photodiodes are summarized in Table 1.

## 4. Conclusions

Hybrid organic–Si B, G, and R image sensor pixels were designed to increase the limits on the photocurrent and the aperture ratio for conventional Si image sensor pixels. They were produced based on B, G, and R hybrid organic–Si photodiodes and four n-MOSFETs. The B, G, and R hybrid organic–Si photodiodes were simply fabricated with typical low-cost, nanoscale-thick B-, G-, and R-sensitive organic small molecules that are used for OLED displays: donor (Coumarin6) and acceptor (C_60) for the B hybrid organic–Si photodiode, donor (DMQA) and acceptor (MePTC) for the G hybrid organic–Si photodiode, and donor (ZnPc) and acceptor (TiOPc) for the R hybrid organic–Si photodiode. Surprisingly, the hybrid organic–Si B, G, and R image sensor pixels presented evidently higher quantum efficiency than the Si image sensor pixel; i.e., there were 17, 11, and 37% increases in the voltage sensing margin difference for hybrid organic–Si B (0.98 V), G (0.93 V), and R (1.15 V) image sensor pixels compared with the Si (0.84 V) image sensor pixel. Since hybrid organic–Si B, G, and R image sensor pixels achieved considerably higher quantum efficiency than the Si image sensor pixel, they likely have the potential to overcome the current photosensitivity limit of the Si image sensor pixel due to the aperture ratio of ~60%; they also offer a convenient image sensor fabrication process using conventional frontside illumination and a low-cost fabrication process. Further research is necessary to produce hybrid organic–Si B, G, and R image sensors with a pixel resolution of 1000 ppi.

## Figures and Tables

**Table 1:** Fabricated structures of B, G, and R hybrid organic–Si photodiodes.

Sensitive Color		Structure
**In our study**	**Blue**	**Al/** **C_60** **/** **Coumarin 6** **/MoO_3_:Al/ITO**
	**Green**	**Al/** **MePTC** **/** **DMQA** **/MoO_3_:Al/ITO**
	**Red**	**Al/** **TiOPc** **/** **ZnPc** **/MoO_3_:Al/ITO**
**NHK**	**Blue**	**ZnO TFT/** **Coumarin 30:C_60** **/Alq3/NTCDA/ITO**
	**Green**	**ZnO TFT/** **NN’** **-** **QA** **/** **Py** **-** **PTC** **/NTCDA/ITO**
	**Red**	**ZnO TFT/** **ZnPc/TiOPc** **/Alq3/ITO**
**Samsung**	**Green**	**ITO/MoOx/** **DM-2,9-DMQA** **/** **SubPc** **/Al**

## Data Availability

Data are contained within the article and Supplementary Materials.

## Supplementary Materials

The following supporting information can be downloaded at: https://www.mdpi.com/article/10.3390/nano14131066/s1, Figure S1: Schematic vertical structure of the CIS, Figure S2: Fabrication process of back-side illumination (BSI) CMOS image-sensor, Figure S3: Transfer characteristics (*I*_D_–*V*_G_) of fabricated four transistors applied *V_th_* implantation, Figure S4: Dependency of dark current of Si photodiode on electrode materials and annealing temperature, Figure S5: Dependency of I–V characteristics and Δ*V_out_* of B-sensitive organic CIS on donor material thickness under light illumination of 20,000 lux, Figure S6: Dependency of I–V characteristics and Δ*V_out_* of G-sensitive organic CIS on donor material thickness under light illumination of 20,000 lux, Figure S7: Dependency of I–V characteristics and Δ*V_out_* of R-sensitive organic CIS on donor material thickness under light illumination of 20,000 lux, Video S1: Video demonstrating dependency of Δ*V_out_* of CIS with green photodiode on light intensity.
