# Supplementary material for: Hybrid Organic–Si C-MOSFET Image Sensor Designed with Blue-, Green-, and Red-Sensitive Organic Photodiodes on Si C-MOSFET-Based Photo Signal Sensor Circuit
Source: Nanomaterials (Basel). 2024 Jun 21;14(13):1066. doi: 10.3390/nano14131066 (PMC11243616; doi:10.3390/nano14131066)
Supplement: Supplementary file 1 [file nanomaterials-14-01066-s001.zip › nanomaterials-3032866-Supplementary Materials.pdf]

nanomaterials

Supplementary Materials

Hybrid Organic–Si C-MOSFET Image Sensor Designed with Blue-, Green-, and Red-Sensitive Organic Photodiodes on Si C-MOSFET-based Photo Signal Sensor Circuit

Ui-Hyun Jeong [1], Joo-Hyeong Park [1,2], Ji-Ho Choi [1], Woo-Guk Lee [3] and Jea-Gun Park [1,4,*]

[1] Department of Electronic Engineering, Hanyang University, Seoul, 04763, Republic of Korea
[2] Samsung Advanced Institute of Technology, 129, Samsung-ro, Yeongtong-gu, Suwon-si, Gyeonggi-do,16677, Republic of Korea
[3] Department of Nanoscale Semiconductor Engineering, Hanyang University, Seoul, 04763, Republic of Korea
[4] Advanced Semiconductor Materials & Devices Development Center, Hanyang University, Seoul, 04763, Republic of Korea
* Correspondence: parkjgl@hanyang.ac.kr; Tel.: +82-2-2220-0234

Supplementary Data

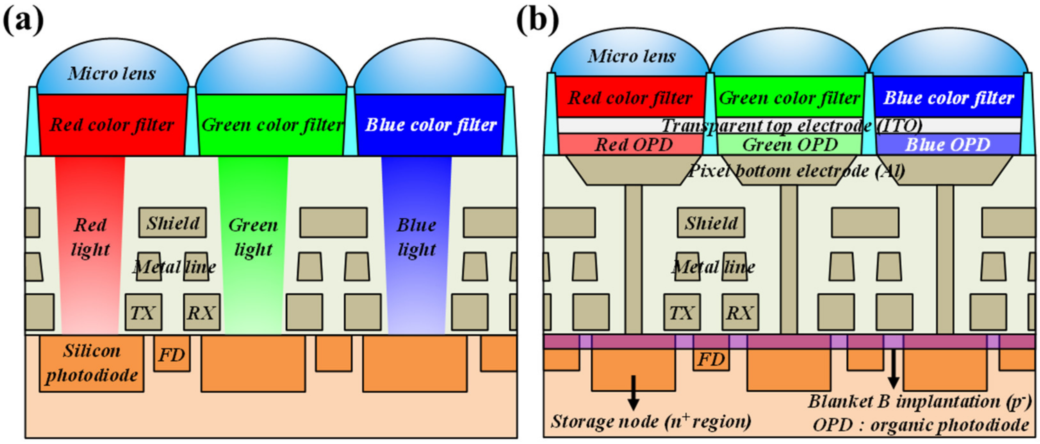

Figure S1. Schematic vertical structure of the CIS: (**a**) conventional CIS and (**b**) proposed hybrid organic CIS with blue, green and red color filter.

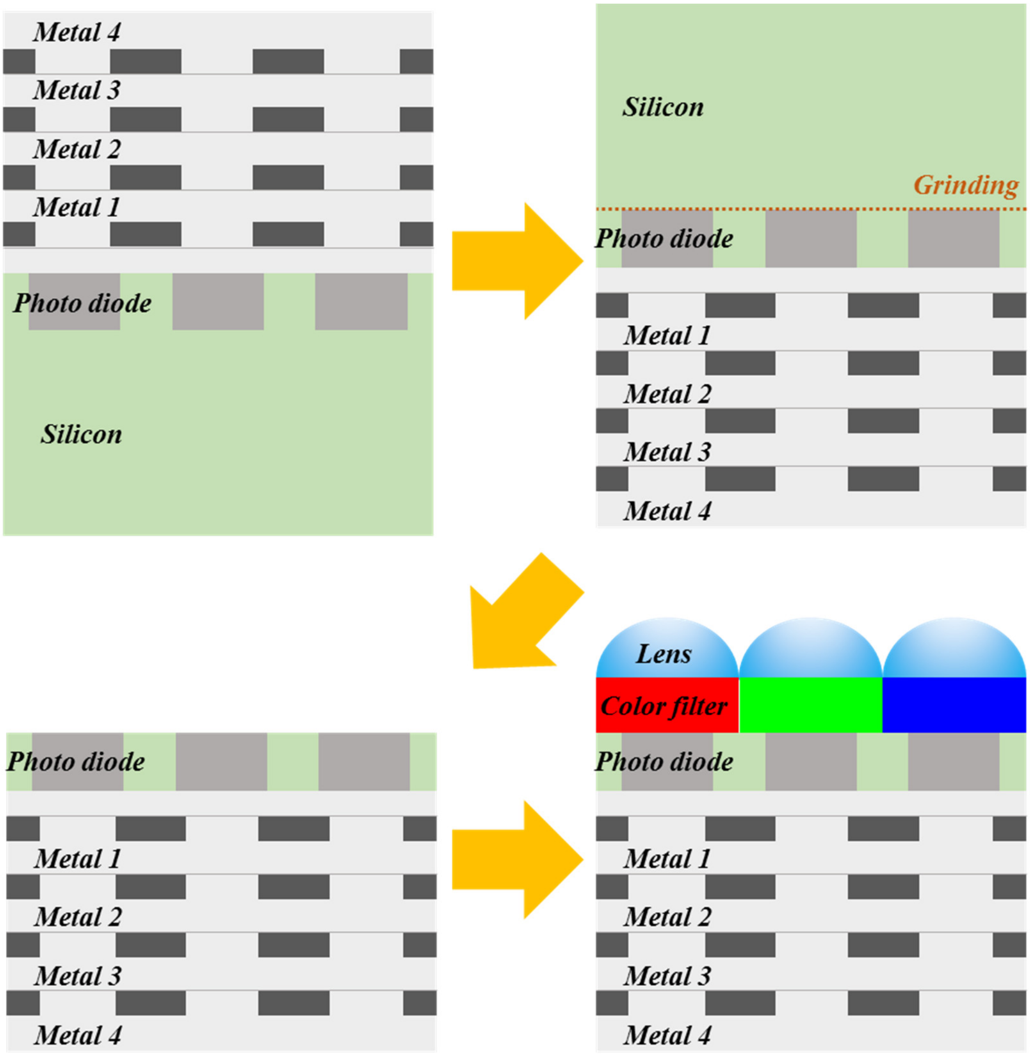

Figure S2. Fabrication process of back–side illumination (BSI) CMOS image–sensor.

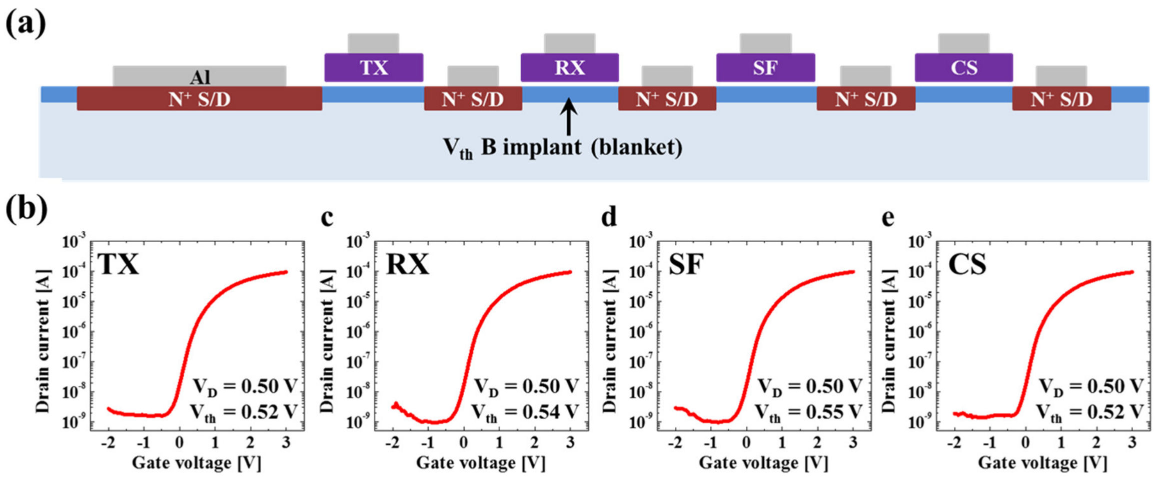

Figure S3. Transfer characteristics (I_D–V_G) of fabricated four transistors applied V_{th} implantation. (**a**) Schematic vertical structure of four transistors, (**b**) I_D–V_G of transfer transistor (*TX*), (**c**) reset transistor (*RX*), (**d**) source follower (*SF*) and (e) current source (*CS*).

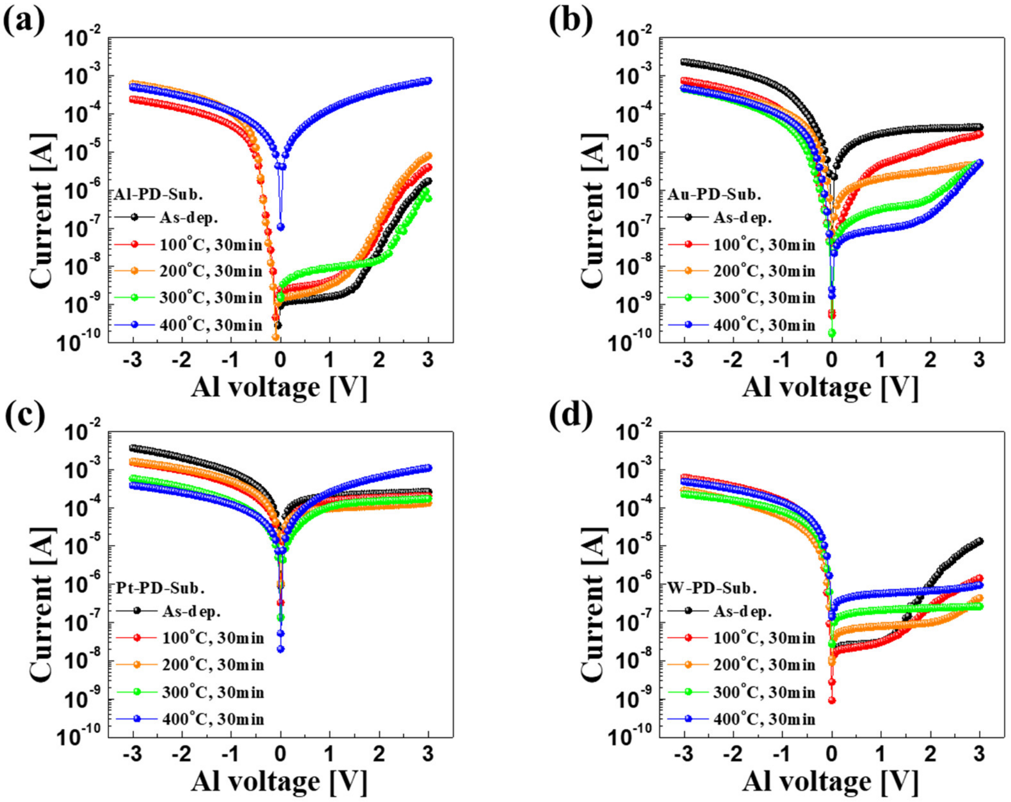

Figure S4. Dependency of dark current of Si photo diode on electrode materials and annealing temperature. (**a**) Al, (**b**) Au, (**c**) Pt and (**d**) W.

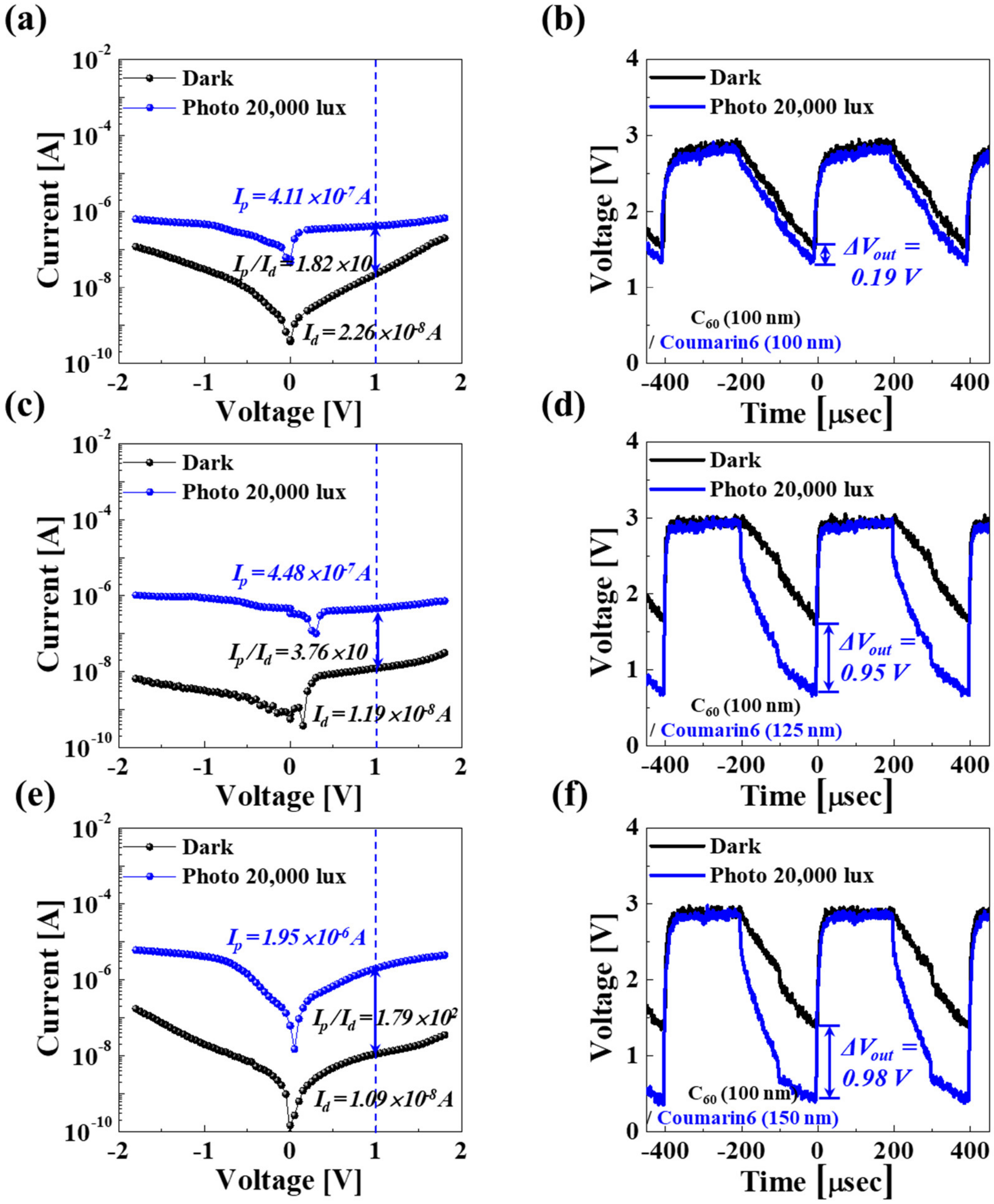

Figure S5. Dependency of *I–V* characteristics and ΔV_{out} of B-sensitive organic CIS on donor material thickness under light illumination of 20,000 lux. (**a**) *I–V*, (**b**) ΔV_{out} of 100–nm Coumarin6, (**c**) *I–V*, (**d**) ΔV_{out} of 125–nm Coumarin6, (**e**) *I–V*, (f) ΔV_{out} of 150–nm Coumarin6.

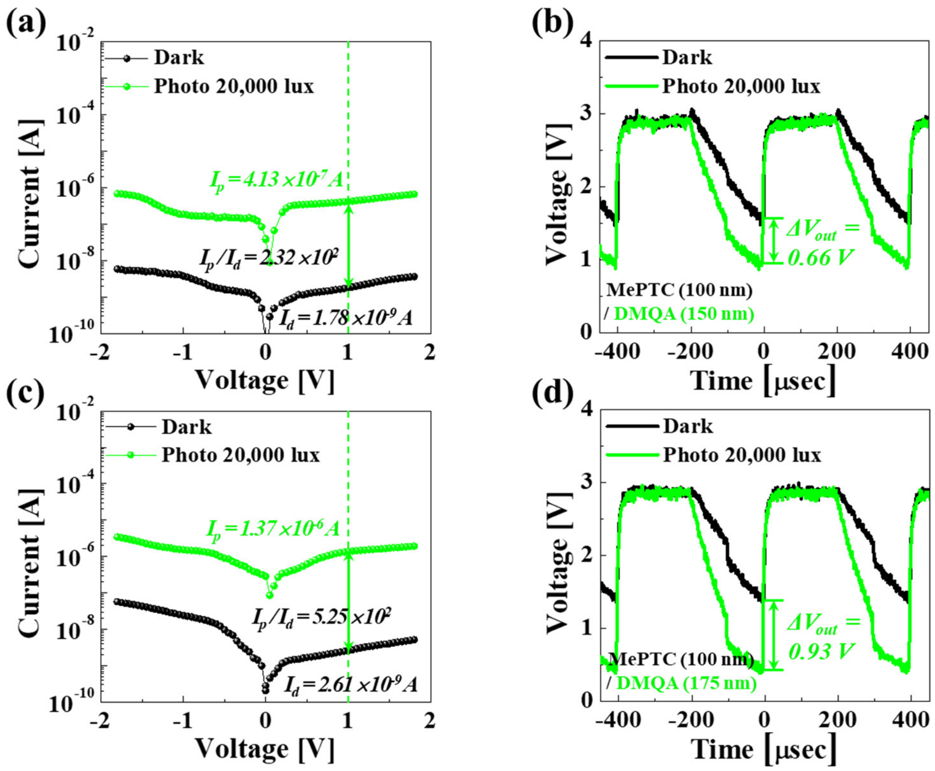

Figure S6. Dependency of *I–V* characteristics and ΔV_{out} of G–sensitive organic CIS on donor material thickness under light illumination of 20,000 lux. (**a**) *I–V*, (**b**) ΔV_{out} of 150–nm DMQA, (**c**) *I–V*, (d) ΔV_{out} of 175–nm DMQA.

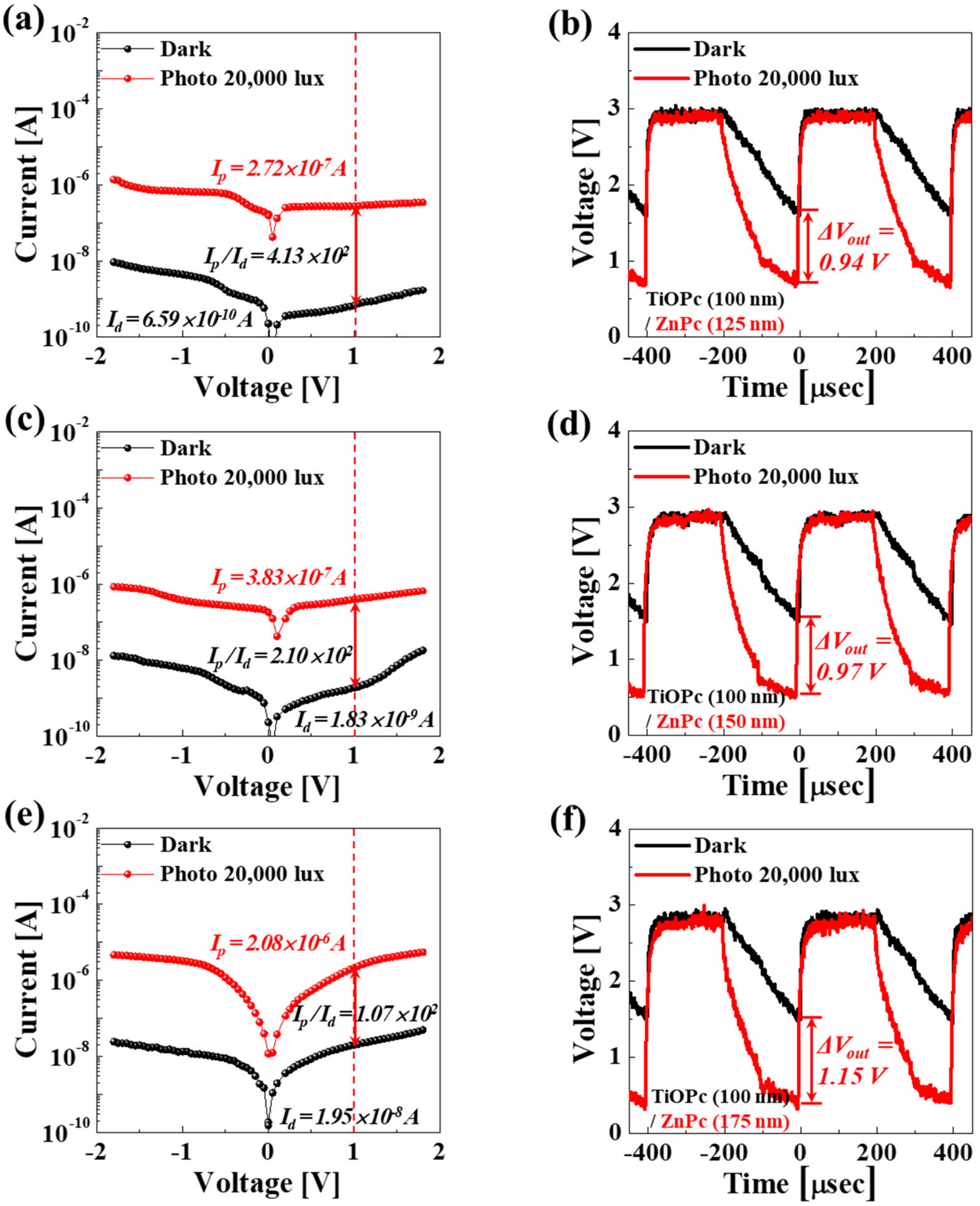

Figure S7. Dependency of *I–V* characteristics and ΔV_{out} of R–sensitive organic CIS on donor material thickness under light illumination of 20,000 lux. (**a**) *I–V*, (**b**) ΔV_{out} of 125–nm ZnPc, (**c**) *I–V*, (**d**) ΔV_{out} of 150–nm ZnPc, (**e**) *I–V*, (**f**) ΔV_{out} of 175–nm ZnPc.
